# IR Studies of the Cu Ions in Cu-Faujasites

**DOI:** 10.3390/molecules24234250

**Published:** 2019-11-22

**Authors:** Łukasz Kuterasiński, Jerzy Podobiński, Dorota Rutkowska-Zbik, Jerzy Datka

**Affiliations:** Jerzy Haber Institute of Catalysis and Surface Chemistry, Polish Academy of Sciences, Niezapominajek 8, 30-239 Krakow, Poland; nckutera@cyf-kr.edu.pl (Ł.K.); ncpodobi@cyf-kr.edu.pl (J.P.); nczbik@cyf-kr.edu.pl (D.R.-Z.)

**Keywords:** IR spectroscopy, Cu sites, CO adsorption, NO adsorption

## Abstract

The properties of Cu ions in dealuminated faujasite-type zeolites (Si/Al = 31) containing 1, 2, and 5 wt.% of Cu were investigated by IR spectroscopy with CO and NO as probe molecules. Cu was introduced by impregnation into zeolites in both protonic (HFAU) and sodium (NaFAU) forms of zeolite. Four kinds of Cu species were found: Cu^+^_exch._, Cu^+^_oxide_, Cu^2+^_exch._ (square, planar, and square pyramidal), and Cu^2+^_oxide_ (CuO). The proportions between these four kinds of Cu depended on the amount of Cu and on the form of zeolite to which Cu was introduced (HFAU or NaFAU). Zeolites with 1 wt.% of Cu introduced to HFAU (denoted as Cu(1)HFAU) contained only Cu^+^_exch._, whereas other forms of Cu were present in zeolites of higher Cu contents. The concentration of Cu^+^_exch._ was determined by quantitative IR studies of CO adsorption. According to the IR results, some Cu ions were situated inside hexagonal prisms and/or cuboctahedra, and were inaccessible to adsorbed molecules. IR studies also evidenced that Cu ions in oxide forms—Cu^+^_oxide_ and Cu^2+^_oxide_ (CuO)—were better electron donors than Cu in exchange positions (Cu^+^_exch._ and Cu^2+^_exch_).

## 1. Introduction

Since the early reports of Ivamoto et al. [1,2,3,4], who observed the activity of CuZSM-5 zeolites in the decomposition of NO into elements, Cu-containing zeolites attracted a lot of attention. Many papers have been published on the decomposition of NO [5,6,7,8,9] and reduction with NH_3_ and hydrocarbons [10,11,12,13,14,15]. Quantum chemical calculations [16,17,18,19] evidenced that the activation of NO was realized by the donation of d-electrons of Cu^+^ to the antibonding π* orbitals of NO. The framework of zeolites played the role of an electron reservoir, and the transfer of electrons from the framework to Cu^+^ enhanced the electrodonor properties of Cu^+^. In other words, the electrodonor properties of Cu^+^ are related to the extent of neutralization of the positive charge of Cu^+^ by framework oxygens.

Cu^+^ ions in zeolites were also found to activate not only N=O bonds but also the multiple bonds in organic molecules, resulting in a significant red shift of the C=C double bond in alkenes (ethene, propene, butenes) [20], a triple C≡C bond in ethyne [21], C=O bonds in acetone and formaldehyde [22,23], as well as the activation of the aromatic ring in benzene [24]. Quantum chemical Density Functional Theory (DFT) calculations showed [25,26,27] that the activation of multiple bonds in these molecules was realized according to a mechanism similar to that of NO activation, i.e., by the donation of electrons from Cu^+^ and the zeolite framework to antibonding π* orbitals of molecules.

As Cu ions in zeolites play such an important role in catalytic processes (mostly in “DeNOx”), their properties were the subject of very extensive studies [28,29,30,31,32,33,34,35,36,37,38,39,40]. Such methods as Infrared Spectroscopy (IR), Electron Spin Resonance (ESR), X-ray Photoelectron Spectroscopy (XPS), Extended X-Ray Absorption Fine Structure (EXAFS), and X-ray Absorption Near Edge Structure (XANES) spectroscopies were applied; Temperature Programmed Desorption (TPD) and Temperature Programmed Reduction (TPR) experiments were also done. Luminescence studies were realized too, and the location of Cu ions in zeolite Y was followed by XRD. These experimental methods provided information about the status and properties of both Cu^2+^ and Cu^+^ ions and their interaction with reactant molecules (mostly with NO).

Recently, it has been found that faujasite-type zeolites containing Cu introduced by impregnation methods were active in reactions in which furfural is reprocessed [41]. This reaction produced mostly furan and methylfuran, which are important substrates for manufacturing other precious chemicals [42,43,44]. Several catalytic processes of furfural hydrogenation with Pd-containing catalysts were elaborated [42,43,44]. Other catalysts not containing noble metals were also used. Some of them were based on zeolites [45,46]. As mentioned, we found [41] that zeolite CuY was active in the transformation of furfural, especially in the production of methylfurfural. It is not excluded that the role of Cu is the activation of hydrogen, which was a substrate in hydrogenation. The earlier IR study of Kazansky and Pidko [47], as well as the results of quantum chemical calculations [47,48], evidenced strong activation of hydrogen by Cu ions in zeolites. It should be noted that Seo and Chon [46] suggested that the role of Pd in the catalysts for furfural hydrogenation was the activation of hydrogen.

Very interesting results were presented by Ordomsky et al. [49] and by Milleto et al. [50], who studied the one-pot production of dimethylether by hydrogenation of CO and CO_2_ over hybrid catalysts: CuZnAl/ZSM-5 and CuZrZn/ferrierite. An important aspect was the problem of deactivation of these catalysts by the migration of Cu to zeolite pores and substitution of protonic sites by Cu ions.

The goal of this study was to use IR spectroscopy to follow the properties of Cu^+^ and Cu^2+^ ions in zeolites of faujasite type to which Cu was introduced by impregnation. As mentioned above, these zeolites were found to be catalysts for furfural conversion. The properties of Cu^+^ were studied with CO and NO as probe molecules. The properties of Cu^2+^ were followed by NO adsorption.

## 2. Results and Discussion

### 2.1. SEM Microscopic and Porosimetric Results

**Figure 1:**
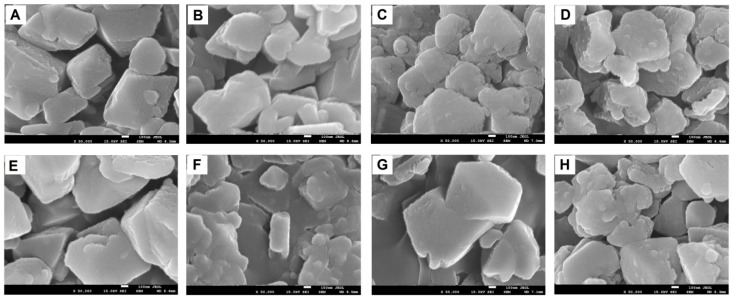
SEM images of the studied samples (50000×): (**A**) HFAU, (**B**) Cu(1)HFAU31, (**C**) Cu(2)HFAU, (**D**) Cu(5)HFAU, (**E**) NaFAU, (**F**) Cu(1)NaFAU, (**G**) Cu(2)NaFAU, and (**H**) Cu(5)NaFAU.

The scanning electron micrographs of the variously modified samples are presented in Figure 1. The faujasite-based catalyst particles are of irregular shape. For the sample pretreated with NaNO_3_ and then modified with Cu species (NaFAU series, Figure 1E–H), the presence of some amorphous phase was found. The deposits of Cu-oxides on external surfaces were not detected.

The results of porosimetric studies, i.e., the volume of micropores measured in N_2_ sorption experiments, are presented in Table 1. The exchange of H in HFAU and production of NaFAU caused some decrease of micropore volume. Most probably, this is due to some loss of crystallinity of the zeolite. The introduction of Cu to both HFAU and NaFAU caused only a small decrease in micropore volume.

### 2.2. OH Groups

**Figure 2:**
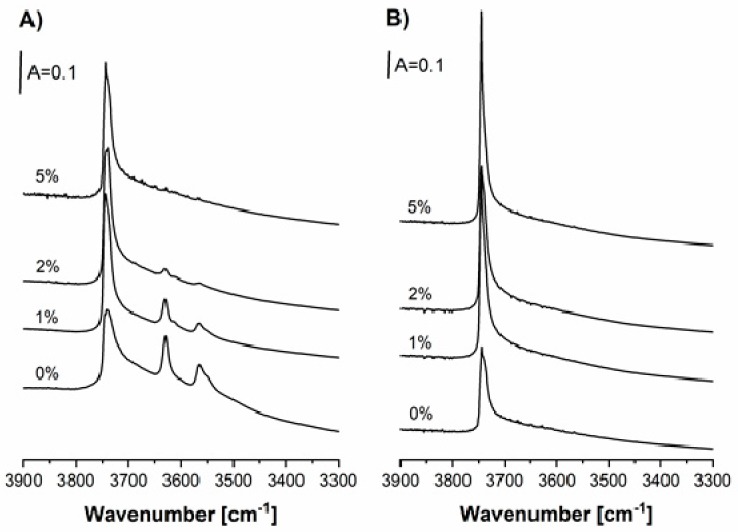
IR spectra of OH groups in zeolites CuHFAU (**A**) and CuNaFAU (**B**) of various Cu contents.

The spectra of OH groups in HFAU and CuFAU zeolites (both CuHFAU and CuNaFAU) with various amounts of Cu are presented in Figure 2A,B. The spectrum of HFAU shows the bands of Si–OH (3740 cm^−1^), Si–O_1_H–Al (3630 cm^−1^), and Si–O_3_H–Al (3565 cm^−1^). The Si–O_1_H–Al groups projecting into supercages were found to be very strongly acidic and homogeneous (all of the same acid strength) [51]. The insertion of Cu caused the decrease of intensity of Si–OH–Al bands. The introduction of 1 and 2% of Cu caused the decrease of the OH bands by ca. 50% and 85%, respectively. The zeolite containing 5% of Cu showed no Si–OH–Al bands. These effects can be attributed to the substitution of protons by Cu ions. It should be noted that the loss of Si–OH–Al groups corresponds to Cu/Al values: 0.37, 0.78, and 1.88. A similar situation was also observed by Ordomsky et al. [49] and by Milleto [50], who concluded that deactivation of the hybrid CuZnAl/ZSM-5 and CuZrZn/ferrierite systems in the production of dimethylether by hydrogenation of CO_2_ and CO was explained by the substitution of protonic sites in zeolites by Cu ions, i.e., by a mechanism similar to that in our study (impregnation).

The NaFAU and CuNaFAU zeolites did not contain Si–OH–Al groups due to substitution of all of the zeolitic protons by Na or Cu ions.

### 2.3. CO Sorption

**Figure 3:**
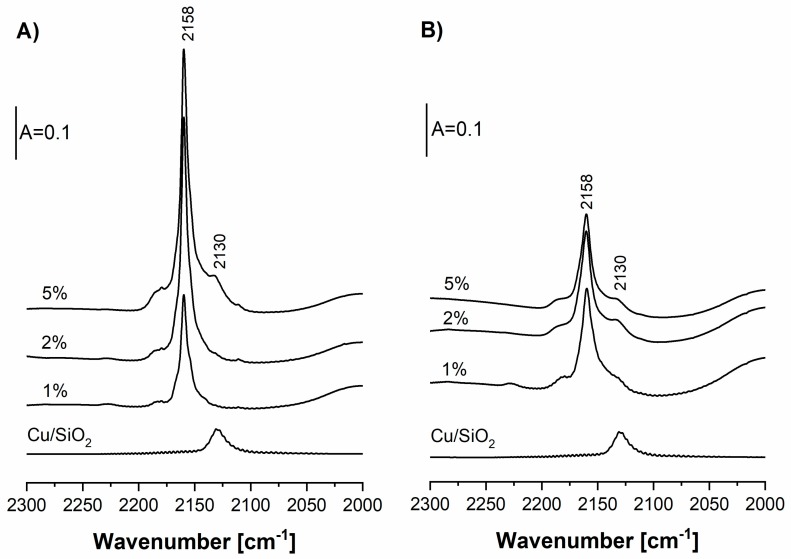
IR spectra of CO interacting at room temperatures with Cu sites in zeolites CuHFAU (**A**) and CuNaFAU (**B**) of various Cu contents. The amounts of CO were sufficient to cover all the Cu sites. The spectra of CO interacting with Cu in Cu/SiO_2_ are also shown.

The spectra of CO sorbed at room temperature in CuHFAU zeolites are presented in Figure 3A. The doses of CO were sorbed until the maximal intensity of the Cu_exch._^+^–CO band at 2158 cm^−1^. The intensity of this band increased if the amount of Cu increased from 1 to 2 wt.%; however, the introduction of 5 wt.% of Cu did not cause further increase of the Cu_exch._^+^–CO band. The band at 2130 cm^−1^, typical of CO interacting with oxide forms of Cu^+^ (Cu_oxide_^+^), is present in the spectrum of CO sorbed in Cu(5)HFAU. The same Cu_oxide_^+^–CO band was also observed if CO was adsorbed on Cu/SiO_2_ (Figure 3A), and was also reported by several authors [52]. The frequency of the Cu_oxide_^+^–CO band (2130 cm^−1^) is lower than that of the Cu_exch._^+^–CO one (2158 cm^−1^) due to the higher degree of neutralization of Cu^+^ by oxygens and the stronger effect of π back donation of d electrons of Cu^+^ to π* antibonding orbitals of CO.

**Figure 4:**
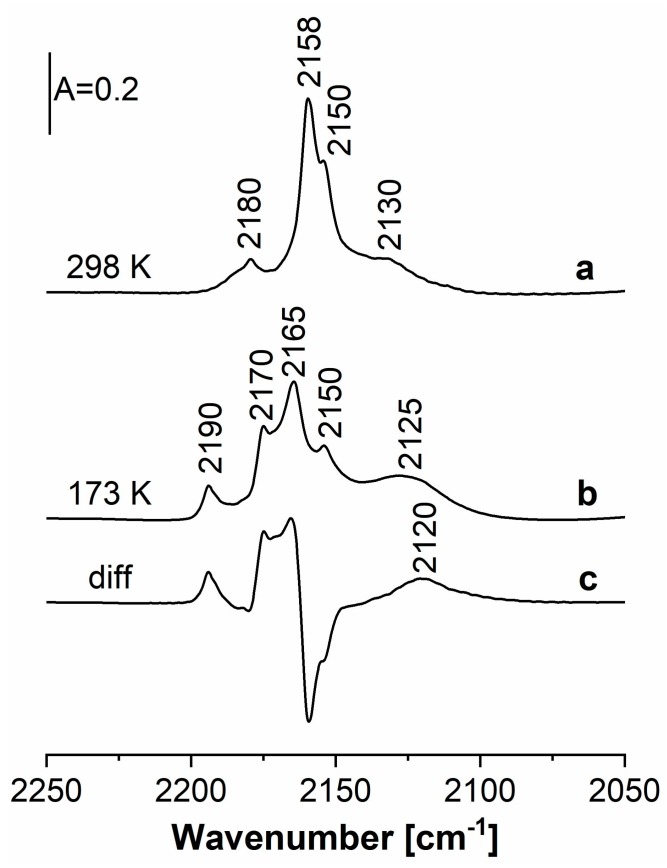
The spectra recorded upon the sorption of CO in the Cu(5)HFAU zeolite at room temperature (**a**), at 170 K (**b**), and difference spectrum (**c** = **b** – **a**).

The spectra recorded upon the sorption at room temperature of excess of CO (sufficient to cover all Cu sites) in Cu(5)HFAU and upon the sorption of excess of CO at 170 K in the same zeolite are presented in Figure 4 (spectra a and b). The bands of Cu_exch._^+^ monocarbonyls (2158 cm^−1^), dicarbonyls (2150 and 2180 cm^−1^), and tricarbonyls (2165, 2170, and 2190 cm^−1^ are present in the spectrum recorded at 170 K). The band of Cu_oxide_^+^–CO at 2125 cm^−1^ is also present. The difference spectrum, c (c = b – a), shows the maxima of the tricarbonyls and the minimum at 2150 cm^−1^, which may be explained by the transformation of dicarbonyls to tricarbonyls. The most important observation concerns the Cu_oxide_–CO band. The maximum of this band is 2130 cm^−1^ in the spectrum recorded at room temperature, but sorption of CO at 170 K produces a new kind of Cu_oxide_^+^-CO species, characterized by a 2120 cm^−1^ band. It may therefore be concluded that two kinds of Cu_oxide_^+^ are present in our CuHFAU with high Cu content (5 wt.%): Greater electron-donating and weakly bonding CO (2120 cm^−1^ CO band) and less electron-donating but more strongly bonding CO (2130 cm^−1^ band).

In our CuHFAU (of Si/Al = 31), the band of Cu_exch_^+^–CO at 2158 cm^−1^ is very narrow (half width ca. 6–8 cm^−1^). The situation is different from that of “typical” zeolite CuY (Si/Al = 2.5), in which two Cu_exch_^+^–CO bands at 2140 and 2160 cm^−1^ were observed [53,54]. An analogous situation concerns Si–OH–Al groups. These hydroxyls were found to be homogeneous in our HFAU [51] and heterogeneous in typical HY (Si/Al = 2.5) [55,56]. This may be explained by the presence of Cu^+^ (or Si–OH–Al), having one Al in the close environment of our FAU (Si/Al = 31), and the presence of Cu^+^ (or Si–OH–Al) and various numbers of Al in the “typical” zeolite Y (Si/Al = 2.5).

**Figure 5:**
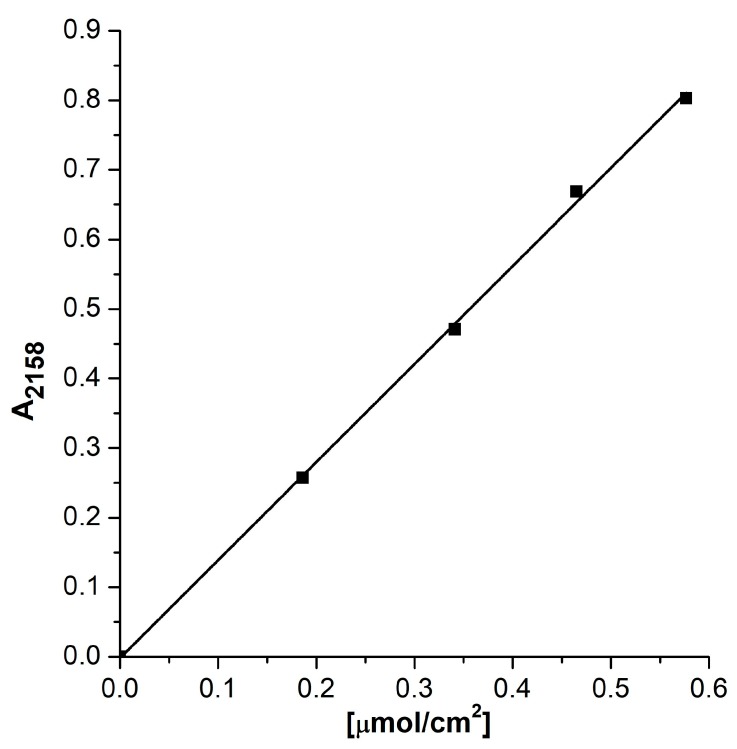
The intensity of the 2158 cm^−1^ band of Cu^+^_exch_.–CO as a function of the amount of CO adsorbed.

The quantitative IR experiments were realized to determine the concentration of the Cu_exch._^+^–CO species. The measured doses of CO were sorbed in zeolite Cu(1)HFAU containing only Cu_exch._^+^. As mentioned above, Cu_oxide._^+^ was absent; Cu^2+^ was also absent, as evidenced by the NO sorption experiments—so, the only adsorption sites were Cu_exch._^+^ (vide infra). A linear increase of intensity of the 2158 cm^−1^ band with the amount of CO sorbed was observed (Figure 5), and the slope of the line is the extinction coefficient of this band. The obtained value of 1.40 cm^2^/µmol was very close to the value obtained in our previous study (1.30 cm^2^/µmol [52]). The concentration of Cu_exch._^+^ was calculated from the maximal intensity of 2158 cm^−1^ and the extinction coefficient of this band. The concentration values are presented in Table 1. For the zeolite Cu(1)HFAU, the concentration of Cu_exch._^+^ (82 µmol/g) was half of the amount of Cu introduced (160 µmol/g, which corresponded to 1 wt.% of Cu). As this zeolite contained only Cu_exch._^+^, the difference between the amount of Cu introduced and Cu reacting with CO may be due to the fact that in zeolite of faujasite type, cations may be located not only in supercages, but also inside hexagonal prisms and cuboctahedra (for example sites S_I_, S_I’_, etc.). Such cations are inaccessible to adsorbed molecules like CO. Such “hidden” positions are preferably occupied by cations, because the stabilization of cations by framework oxygens in these sites is the most effective.

Zeolite Cu(2)FAU contains a higher concentration of Cu_exch._^+^ than that of Cu(1)FAU; however, this concentration is still lower than the amount of Cu introduced (Table 1). This difference can also be explained by the location of some Cu in sites inaccessible to CO, but also by the presence of some Cu^2+^ in this zeolite (vide infra). The increase of Cu content from 2 to 5% did not cause a further increase of the amount of Cu_exch._^+^. It seems that in this zeolite, most of the Cu is in the form of Cu_oxide_^+^ and Cu^2+^. It is also possible that in zeolites of high Cu contents, some Cu species are in big agglomerates that are not accessible to adsorbed molecules.

As mentioned, in zeolites Cu(1)HFAU and Cu(2)HFAU, only half of Cu^+^ is accessible to CO molecules. The location of Cu cations in the structure of zeolite CuY was the subject of detailed studies by Palomino et al. [29]. XRD studies by these authors evidenced that if CO was adsorbed at a very low temperature (80 K), tricarbonyls Cu^+^(CO)_3_ were formed, and the strong interaction with three CO ligands caused the migration of some Cu^+^ from the positions inside cuboctahedra to S_II_ sites accessible to CO. In our case, CO was sorbed at room temperature and Cu_exch._^+^–CO monocarbonyls were formed. It seems that the interaction of Cu_exch._^+^ with one CO ligand is not strong enough to cause the migration of Cu^+^.

The spectra of CO sorbed in CuNaFAU zeolites are presented in Figure 3B. The amount of CO was sufficient to react with all Cu sites accessible to CO. In this case, the intensity of the Cu_exch._^+^–CO (2158 cm^−1^) band was distinctly lower than that in CuHFAU. The same conclusion was obtained in quantitative studies (Table 1). The concentration of Cu_exch._^+^ was ca. 30 µmol/g and was independent of Cu content. These results evidence that the ionic exchange of Cu in zeolite NaFAU was much less efficient than in the case of HFAU. This may be explained by considering the exchange equilibrium: zeol-H + Cu(NO_3_)_2_→zeol-Cu + HNO_3_. HNO_3_ is removed during the calcination which followed ionic exchange, which shifts the exchange equilibrium towards the production of zeol-Cu. On the other hand, for the Na-form of the zeolite, the ionic exchange can be represented by the formula: zeol-Na + Cu(NO_3_)_2_→zeol-Cu + NaNO_3_. NaNO_3_ was not removed during calcination and shifts the exchange equilibrium back towards zeol-Na. Cu(NO_3_)_2_, which was not consumed during ion exchange, decomposed during calcination, forming Cu oxide forms. The band of Cu_oxide_^+^–CO at 2130 cm^−1^ was also present, indicating that some Cu^+^ was in the form of oxide. Cu^2+^ was also detected in NO sorption experiments (vide infra).

### 2.4. NO Sorption

**Figure 6:**
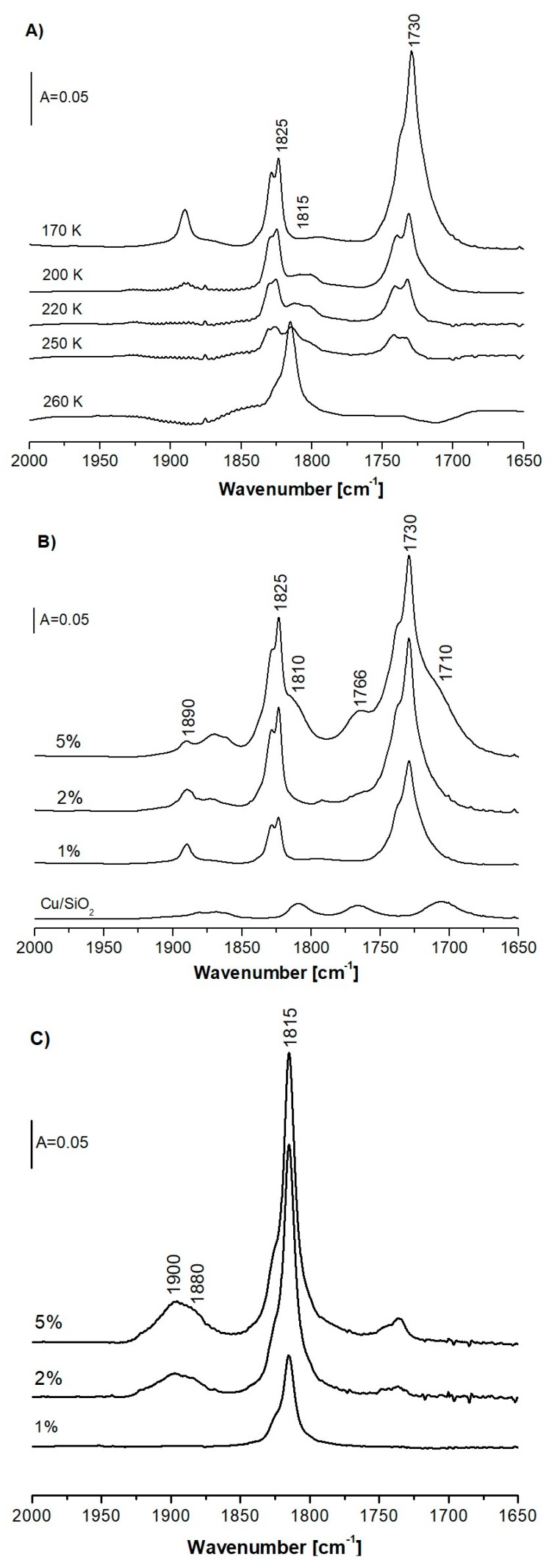
(**A**)—The spectra recorded upon the sorption of NO in zeolite Cu(1)HFAU at 170 K and upon heating of zeolite with NO to 200, 220, 250, and 260 K. (**B**), (**C**)—The spectra of NO sorbed in CuHFAU zeolites at various Cu contents at 170 K (**B**) and at room temperature (**C**).

CO is a very useful probe molecule for Cu^+^ sites; however, NO is more helpful for characterization of Cu^2+^. We sorbed NO in CuHFAU and CuNaFAU at a low temperature (ca. 170 K), and the zeolite with adsorbed NO was heated up to room temperature. The spectra are presented in Figure 6A. The spectrum recorded at 170 K shows two bands of Cu_exch._^+^ (NO)_2_ dinitrosyls at 1730 and 1825 cm^−1^. The band at 1890 cm^−1^ may be attributed to NO interacting with Lewis acid sites (extraframework Al), which is present in all of our zeolites (this band is also present in the spectra of NO sorbed in HFAU without Cu—spectrum not shown). The heating of the zeolite with NO causes the substitution of dinitrosyl bands with mononitrosyl ones at 1815 cm^−1^ and desorption of NO from Lewis sites.

The spectra of NO sorbed at ca. 170 K in CuHFAU zeolites of various Cu content are presented in Figure 6B. All of the spectra show the bands of Cu_exch._^+^ (NO)_2_ dinitrosyls at 1730 and 1825 cm^−1^, as well as a new band at 1766 cm^−1^, the intensity of which increases with Cu content. The shoulders at 1710 and 1810 cm^−1^ are seen in the case of zeolite Cu(5)HFAU at the highest Cu content. In order to identify the species responsible for the 1710, 1766, and 1810 cm^−1^ bands, NO was adsorbed at ca. 170 K on Cu/SiO_2_ not containing Cu_exch._^+^ (see Figure 6B). The 1710, 1766, and 1810 cm^−1^ bands are present. It may be concluded that the band at 1766 cm^−1^ may be attributed to Cu_oxide_^+^–NO mononitrosyls and the bands at 1710 and 1810 cm^−1^ are due to Cu_oxide_^+^(NO)_2_ dinitrosyls formed on Cu_oxide_^+^. The presence of Cu_oxide_^+^ in Cu(5)FAU and Cu/SiO_2_ was evidenced in CO sorption experiments (Figure 3A). The frequency of both mononitrosyl and dinitrosyl bands for Cu_oxide_^+^ is lower than for Cu_exch._^+^. This may be explained by the partial neutralization of Cu^+^ charge by oxygens in oxide-like clusters and more effective π back donation of d electrons of Cu^+^ to the π* antibonding orbitals of NO. The spectra of NO sorbed in zeolites CuHFAU show also a band at 1890 cm^−1^ of NO interacting with Lewis acid sites, and the broad, weak band in the region of 1850–1900 cm^−1^ is attributed to NO interacting with Cu^2+^. The Cu^2+^–NO bands are also clearly seen in the spectra recorded at higher temperatures (ca. 260 K) presented in Figure 6C. The mononitrosyl band at 1815 cm^−1^, the intensity of which increases with Cu content, is present in the spectra recorded at 260 K. Palomino et al. [29] observed two bands of mononitrosyl Cu^+^_exch._–NO in CuY (Si/Al = 2.7) at 1792 and 1815 cm^−1^, attributed to the interaction of NO with Cu^+^ in sites S_II_* and S_II_, respectively. In our CuHFAU of much higher Si/Al (Si/Al = 31) and of much lower Cu content, the sites of Cu_II_* are not occupied; we observed only the 1815 cm^−1^ band of NO interacting with Cu in S_II_.

The broad band of NO interacting with Cu^2+^ at 1850–1900 cm^−1^ is present in zeolites containing 2 and 5 wt.% of Cu. The amount of Cu^2+^ increases with Cu content, and it is absent in zeolites containing 1 wt.% of Cu. The nature of Cu^2+^ in zeolites was the subject of several studies which used NO as a probe molecule. According to Ziolek et al. [57], as well as other authors [58,59], the bands at ca. 1880 and 1900 cm^−1^ were assigned to Cu^2+^ in square planar and square pyramidal coordinations with four oxygens, i.e., to Cu^2+^ which is less positive and more positive, respectively. The Cu^2+^–NO maximum at ca. 1860 cm^−1^ was assigned to CuO [60]. Palomino et al. [29] also reported the presence of narrow bands at 1923 and 1955 cm^−1^, attributed to Cu^2+^ in trigonal planar (S_II_*) and trigonal pyramidal (S_II_) oxygen coordinations. In our CuHFAU (Figure 6B,C), these bands are absent, indicating that Cu^2+^ is not situated in either the S_II_ or S_II_* position. It should be noted that S_II_ positions are occupied by Cu_exch_^+^. The 1850–1900 cm^−1^ band is relatively broad; it may be supposed that it contains both 1880 and 1900 cm^−1^ components attributed to Cu^2+^ in square planar and pyramidal coordinations. These sites are S_III_ ones. The spectra of NO sorbed in Cu-zeolites at low temperatures (Figure 6A,B) show a band at 1860–1870 cm^−1^, the intensity of which increases with Cu content. It is possible that this band may be attributed to NO interacting with CuO [60]. Cu^2+^ in CuO bonds with NO very weakly, and NO desorbs upon increase of temperature.

**Figure 7:**
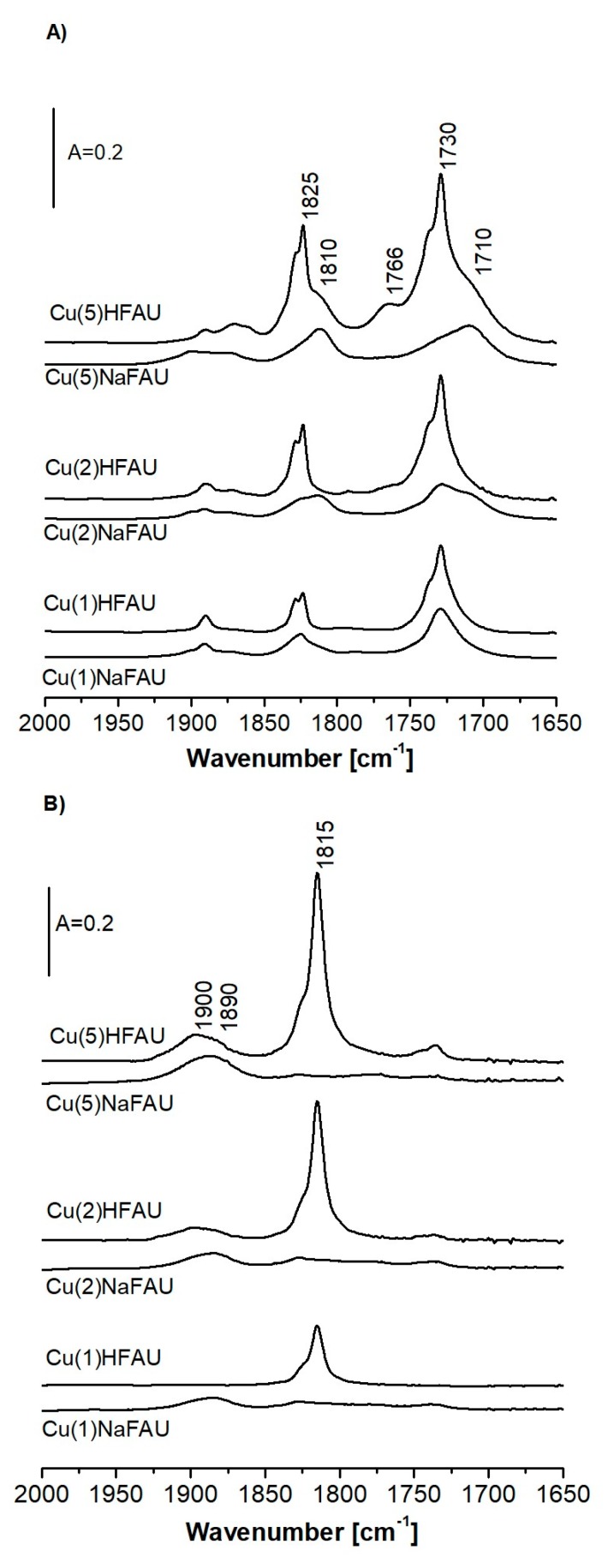
The spectra of NO sorbed at 170 K in zeolites CuHFAU (**A**) and CuNaFAU (**B**) of various Cu contents.

The spectra of NO sorbed over CuNaFAU zeolites as well as in CuHFAU are presented in Figure 7A,B. In CuNaFAU zeolites, most of the Cu is in oxide form (dinitrosyl maxima at 1710 and 1810 cm^−1^), which agrees with the results of CO sorption (Figure 3B). Very weak bands of Cu_exch._^+^(NO)_2_ are present only in Cu(1)NaFAU and Cu(2)NaFAU. The spectra of NO sorbed at room temperature (Figure 7B) show the bands of NO interacting with Cu^2+^ at 1850–1900 cm^−1^. The comparison of spectra of NO interacting with Cu^2+^ sites in CuNaFAU and CuHFAU suggests that CuNaFAU zeolites contain a higher contribution of Cu^2+^ in square planar positions. It is also possible that these zeolites contain CuO.

**Figure 8:**
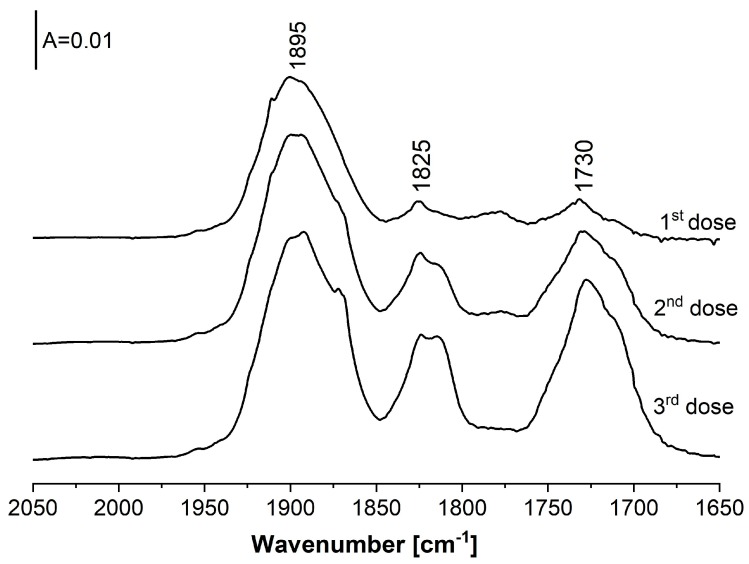
The spectra recorded upon the sorption of increasing amounts of NO at 170 K in zeolite Cu(5)NaFAU.

The spectra recorded upon the sorption of three doses of NO at 170 K in zeolite Cu(5)NaFAU are presented in Figure 8. The spectra evidence that NO reacts firstly with Cu^2+^ (the band around 1895–1900 cm^−1^ appears), and with Cu^+^ in the next order (the Cu^+^(NO)_2_ dinitrosyl bands at 1730 and 1825 cm^−1^ appear). It may therefore be concluded that Cu^2+^ binds NO more strongly than Cu^+^.

## 3. Materials and Methods

### 3.1. Catalyst Preparation

A protonic form of faujasite-type zeolite of Si/Al = 31 was supplied by Zeolyst company (CBV 760—denoted as HFAU, Farmsum, The Netherlands). It was dealuminated by steaming and acid treatment. Two series of Cu-containing zeolites were prepared. One series was obtained by impregnation of HFAU with 0.5 M Cu(NO_3_)_2_ solution, and the zeolites containing 1, 2, and 5 wt.% Cu were denoted as Cu(1)HFAU, Cu(2)HFAU, and Cu(5)HFAU, respectively. In order to prepare the second series, the commercial HFAU was first transformed into Na-form by fourfold exchange with 0.5 M NaNO_3_, followed by washing in distilled water. NaFAU was subsequently impregnated with 0.5 M Cu(NO_3_)_2_, and zeolites containing 1, 2, and 5 wt.% of Cu, which were denoted as Cu(1)NaFAU, Cu(2)NaFAU, and Cu(5)NaFAU, respectively, were obtained. The amounts 1, 2, and 5% of Cu correspond to Cu/Al = 0.38, 0.76, and 1.88. All samples were dried at 390 K and then calcined at 770 K.

### 3.2. Chemical Analysis

Si, Al, and Na contents in the parent zeolite were determined by Inductively Coupled Plasma Optical Emission Spectroscopy (ICP OES) on an Optima 2100DV (PerkinElmer, Arkon, OH, USA) instrument. In order to determine the composition of the zeolite, 70–80 mg of a zeolite sample was treated with a mixture of 0.3 mL HF and 3 mL of concentrated HCl in a Teflon vessel for 24 h. After the dissolution of the zeolite, the liquid was diluted to 50 mL and Si, Al, and Na amounts (45.7, 1.48, and 0.016 wt.% respectively) were determined by ICP OES spectroscopy. The accuracy of measurement was ca. 5–10%. The Si/Al value (Si/Al = 31) was calculated from analysis of the results.

### 3.3. Porosimetric Studies

The sorption of nitrogen was followed at 77 K using a Quantachrome apparatus (Nova, Hook, UK). Before experiments, a sample was evacuated in situ in the Micromeritics cell at 670 K for 12 h. Micropore volume and surface was determined by applying the *t*-plot method. The accuracy of these measurements was ca. 10%.

### 3.4. Microscopic Studies

The SEM measurements were carried out using a Jeol JSM-7500F scanning electron microscope (JEOL, Tokyo, Japan) equipped with the X-ray energy dispersive (EDS) system INCAPentaFETx3. The samples were dried for 24 h and coated with chromium (20 nm) directly before measurements.

### 3.5. IR Studies

Prior to IR experiments, zeolites were evacuated in situ in an IR cell at 720 K for 1 h. The spectra were recorded with a NICOLET 6700 spectrometer (Thermo Scientific, Cambridge, MA, USA) with a spectral resolution of 1 cm^−1^. CO and NO (Air Products) were used as probe molecules. The adsorption of CO was performed at room temperature and at ca. 170 K. Adsorption of NO was also done at ca. 170 K, and zeolites with adsorbed NO were heated to room temperature. The spectra were each recorded at 10 K.

## 4. Conclusions

Totals of 1, 2, and 5 wt.% of Cu were introduced by impregnation into HFAU and NaFAU zeolites of Si/Al = 31. The status and properties of Cu were studied by IR spectroscopy with CO and NO as probe molecules.

Both Cu^+^ and Cu^2+^ were found in the zeolites. Cu^+^ was found in the form of exchange cations (Cu^+^_exch._) and in oxide form (Cu^+^_oxide_). The experiments of CO sorption at various temperatures evidenced that two kinds of Cu^+^_oxide_ with various electrodonor properties are present in the studied systems. Cu^2+^ was found both in the form of CuO and Cu^2+^ in square planar and square pyramidal coordinations with framework oxygens. The proportion between various forms of Cu depends on both the amount of Cu and the form of the FAU-type zeolite (HFAU or NaFAU). In CuHFAU containing 1 wt.% of Cu, all of the Cu species were in the form of Cu^+^_exch._ neutralizing AlO_4_^−^. Quantitative IR studies of CO sorption evidenced that half of Cu^+^_exch._ was inside supercages which were accessible to reactant molecules, and half inside cuboctahedra and hexagonal prisms. The increase of Cu content caused an increase in the amount of Cu^+^_exch.,_ but still, some Cu was hidden inside small cages. Zeolites of higher Cu content also contained Cu^+^_oxide_ and Cu^2+^ in the form of Cu^2+^_exch._ and of CuO. CuNaFAU zeolites demonstrated a much lower contribution of Cu^+^_exch._ And a higher contribution of Cu^+^ and Cu^2+^ in oxide forms.

## Figures and Tables

**Table 1:** Micropore volume and the concentration of Cu sites in zeolites.

	% Cu	Micropore Volume [cm^3^/g]	Concentration of Cu Sites [µmol/g]	
			Introduced	Reacting with CO
CuHFAU	0	0.260		
	1	0.218	160	80
	2	0.222	320	160
	5	0.213	800	170
CuNaFAU	0	0.154		
	1	0.126	160	30
	2	0.129	320	30
	5	0.131	800	30
